# Anesthesiologists’ and surgeons’ perceptions about routine pre-operative testing in low-risk patients: application of the Theoretical Domains Framework (TDF) to identify factors that influence physicians’ decisions to order pre-operative tests

**DOI:** 10.1186/1748-5908-7-52

**Published:** 2012-06-09

**Authors:** Andrea M Patey, Rafat Islam, Jill J Francis, Gregory L Bryson, Jeremy M Grimshaw

**Affiliations:** 1 Centre for Practice-Changing Research, Ottawa Hospital Research Institute – General Campus, Smyth Road, Ottawa, ON, Canada; 2 Faculty of Medicine, University of Ottawa, Smyth Road, Ottawa, ON, Canada; 3 Health Psychology Group and Health Services Research Unit, University of Aberdeen, Foresthill campus, Aberdeen, UK; 4 Department of Anesthesia, The Ottawa Hospital Civic Campus, Ottawa, ON, Canada

**Keywords:** Routine pre-operative testing, Anesthesia management, Anesthesiologists, Surgeons, Chest x-rays, Electrocardiograms, Theoretical domains framework, Semi-structured interviews, Content analysis, Social, Professional role and identity, Social influence

## Abstract

**Background:**

Routine pre-operative tests for anesthesia management are often ordered by both anesthesiologists and surgeons for healthy patients undergoing low-risk surgery. The Theoretical Domains Framework (TDF) was developed to investigate determinants of behaviour and identify potential behaviour change interventions. In this study, the TDF is used to explore anaesthesiologists’ and surgeons’ perceptions of ordering routine tests for healthy patients undergoing low-risk surgery.

**Methods:**

Sixteen clinicians (eleven anesthesiologists and five surgeons) throughout Ontario were recruited. An interview guide based on the TDF was developed to identify beliefs about pre-operative testing practices. Content analysis of physicians’ statements into the relevant theoretical domains was performed. Specific beliefs were identified by grouping similar utterances of the interview participants. Relevant domains were identified by noting the frequencies of the beliefs reported, presence of conflicting beliefs, and perceived influence on the performance of the behaviour under investigation.

**Results:**

Seven of the twelve domains were identified as likely relevant to changing clinicians’ behaviour about pre-operative test ordering for anesthesia management. Key beliefs were identified within these domains including: conflicting comments about who was responsible for the test-ordering (Social/professional role and identity); inability to cancel tests ordered by fellow physicians (Beliefs about capabilities and social influences); and the problem with tests being completed before the anesthesiologists see the patient (Beliefs about capabilities and Environmental context and resources). Often, tests were ordered by an anesthesiologist based on who may be the attending anesthesiologist on the day of surgery while surgeons ordered tests they thought anesthesiologists may need (Social influences). There were also conflicting comments about the potential consequences associated with reducing testing, from negative (delay or cancel patients’ surgeries), to indifference (little or no change in patient outcomes), to positive (save money, avoid unnecessary investigations) (Beliefs about consequences). Further, while most agreed that they are motivated to reduce ordering unnecessary tests (Motivation and goals), there was still a report of a gap between their motivation and practice (Behavioural regulation).

**Conclusion:**

We identified key factors that anesthesiologists and surgeons believe influence whether they order pre-operative tests routinely for anesthesia management for a healthy adults undergoing low-risk surgery. These beliefs identify potential individual, team, and organisation targets for behaviour change interventions to reduce unnecessary routine test ordering.

## Background

Pre-operative tests are ordered to aid in the management of surgical patients. These pre-operative tests provide information about the function of the biological systems that may not be directly affected by the surgical condition, but may be relevant to the perioperative course [1]. However, many pre-operative tests are routinely ordered for apparently healthy patients without any clinical indication, and the subsequent test results are rarely used [2]. In addition, unnecessary testing may lead physicians to pursue and treat borderline and false-positive laboratory abnormalities [3]. A randomized control study (RCT) of over 19,000 cataract patients found no benefit to routine pre-operative medical testing when stratified according to age, gender, or race of the patient, and most abnormalities in laboratory values could be predicted from patient’s history and physical exam [4]. Further, Chung *et al.* conducted an RCT of routine pre-operative testing in 1,057 ambulatory patients where one arm received pre-operative tests ordered according to the Ontario Pre-operative Testing Grid [5] and the other received no pre-operative tests routinely ordered for anesthesia management [6]. They reported no significant difference between rates of perioperative adverse events and the rates of adverse events 30 days after surgery between groups [6].

The Canadian Anesthesiologists’ Society (CAS) has published guidelines to aid pre-admission teams about the appropriateness of certain tests prior to surgery [7]. They advocate that investigations should not be ordered on a routine basis, but should be based on the patient’s health status, drug therapy, and with consideration to the proposed surgical intervention [7]. However, in a study conducted by Hux *et al.* that looked at patterns of pre-operative chest x-rays and electrocardiogram—two tests commonly ordered routinely for anesthesia management—use in Ontario surgical patients, they reported considerable variation in testing rates in low-risk procedures across the province as well as within institutions [8]. In 50 Ontario hospitals, for low-risk (outpatient) procedures (cystoscopy, cataract removal, laparoscopic cholescystectomy, hysterectomy), hospital-specific rates of patients receiving chest x-ray, electrocardiogram, or both ranged from less than 1% to 98% [8]. These findings suggest that factors other than evidence of patient benefit may influence test ordering behaviour.

Failure to convert recommendations into practice is often not related to the content or quality of the guideline but to difficulties in changing established behaviours of the clinicians and institutions [9]. Canadian surgical patients encounter a number of healthcare providers responsible for their experience in the healthcare system including the family physician writing the referral, the attending surgeon, the attending anaesthesiologist, nursing staff, and the myriad of professionals in the pre-admissions clinic. Translating guidelines into clinical practice is notoriously difficult when one healthcare professional has decision-making autonomy; it can be even more so when a group of professionals are responsible, as is the case with pre-operative test ordering. While the guidelines for pre-operative testing are recommendations for anaesthesiologists, other clinicians can and do order pre-operative tests. Bryson reported that surgeons were responsible for 80% of the test ordering that were in non-compliance with the Ontario Pre-operative Testing Grid at the Ottawa Hospital [10]. When many groups of professionals can be the potential target of behaviour change interventions, understanding the thoughts and opinions of the key clinical decision makers about the behaviour in question becomes important. However, much of the work examining health practitioner behaviour change has, to date, been largely atheoretical [11-14]. Using theory for identifying determinants of behaviour and selecting interventions can increase the likelihood of the complex interventions being appropriate [15]. Empirically-supported theories of behaviour change may thus inform attempts to change test-ordering behaviour. Establishing a better theoretical understanding of healthcare professional behaviours and their perceptions of team behaviours may increase the likely success of interventions to change clinical practice.

Psychological theories have long been used to understand, predict, or generate behaviour change in healthcare providers [11,16-19]. Commonly, researchers have tested a single or small number of theories. As a result, only a small range of the potential influences on behaviour are tested. Such studies may be uninformative if the key determinants of the behaviour under question are not represented in the tested theories. Currently, there is little rationale to guide choice of potentially relevant theories. In an attempt to address these problems, Michie *et al*. [20] applied a systematic consensus approach to develop a framework grounded in psychological theory that simplifies theories relevant to behaviour change. The consensus identified 12 theoretical domains from 33 theories and 128 constructs that may explain health-related behaviour change. The Theoretical Domains Framework (TDF) can be used to inform the choice of potential behaviour change techniques to develop interventions as well as to investigate determinants of behaviour [20].

In this study, we used the TDF to systematically examine the beliefs of anaesthesiologists and surgeons about the use of pre-operative testing routinely ordered for anesthesia management in healthy patients undergoing low-risk surgical procedures. This article is one in a series of articles documenting the development and use of the Theoretical Domains Framework (TDF) to advance the science of implementation research [21-24]. Greater detail about the TDF can be found in the introductory article of this series [23].

## Methods

### Design

This was an interview study using semi-structured interviews with anaesthesiologists and surgeons.

### Participants

Participants were selected using a snowball sampling strategy supplemented with purposive sampling techniques. The snowball sampling was used to identify key informants likely to be knowledgeable about the topic being discussed. We identified two or three individuals who would be willing to participate and subsequently requested that they identify additional two individuals they believed would provide valuable information regarding preoperative test ordering practice for anesthesia management.

The criteria used to select the potential interviewees were that they cared for individuals for whom the behaviour under investigation is relevant and were representative of community and academic hospitals. Additionally, in an attempt to avoid premature saturation, we asked the participants to recommend additional anesthesiologists with differing opinions. Because anesthesiologists in Ontario may staff both the pre-admission clinics and the operation rooms on a rotating basis, they could provide their experience from both roles when we asked questions about ordering and reviewing tests. While we had originally planned on only interviewing anesthesiologists (as they are primarily responsible for ordering tests relevant to anesthesia management), surgeons were added to the sampling after six interviews with anesthesiologists. It became apparent after these six interviews the strong influence surgeons had on the test ordering practice of the anesthesiologists and we decided to include them in the study. Our sampling criteria for the surgeons was similar to that of the anesthesiologist in that the surgeons cared for individuals for whom the behaviour under investigation is relevant, however we did not purposively sample by different surgical subspecialty. We continued to add both anesthesiologists and surgeons and used the concept of data saturation to determine when we no longer needed to continue interviewing. In other words, we conducted interviews with each group until no new information was being offered [25], which occurred after 16 interviews (anesthesiologists and surgeons).

### Interview topic guide

The behaviour of interest was ordering of pre-operative tests for anesthesia management (chest x-ray (CXR) and electrocardiographs (ECG)) in a healthy patient having low-risk surgery (knee arthroscopy, laparoscopic cholescystectomy, or cataract removal, lens replacement, and similar type surgeries). Healthy patients were defined as those patients without any co-morbidity or additional medical conditions that could complicate anesthesia management and perioperative care other than the ailment for which surgery is required. An interview topic guide was developed based on the Theoretical Domains Framework to elicit beliefs about each domain for the behaviour, and obtain greater detail about the role of the domain in influencing the behaviour [18]. With advice of a content expert in the field of anesthesia (GLB), the guide was adapted from the original framework [20] to be appropriate to the specific behaviour and clinical context. Questions about ordering and reviewing tests for anesthesia management were included in the interview guide because these two behaviours form part of a continuum; reviewing tests typically occurs on the day of surgery, several days after the tests were originally ordered. We wanted to determine if and why clinicians ordered tests for other clinicians but may not review tests ordered for them on the day of surgery. After pilot testing with two anesthesiologists, wording of some questions from the original TDF had to be modified to fit the context of the behaviour. Subsequent piloting with a further two anesthesiologists resulted in additional wording changes to enhance clarity of one question (See Additional file 1 for Interview Topic Guide).

### Procedure

Participants were contacted in writing and invited for an interview at a time convenient to them. All interviews (conducted by AMP) were conducted by phone or in person. The interviews were digitally recorded and lasted between 14 and 46 minutes. The recordings were transcribed and anonymised.

### Analysis

Two researchers (AMP, RI) coded interview participants’ responses into the relevant theoretical domains. Two pilot interviews were used to formulate a coding strategy. The first pilot interview was coded by two researchers in tandem to develop the coding strategy, and the second was used to ensure the two coders were comfortable with the strategy developed from the first. Subsequent coding of the remaining interviews was completed independently and Fleiss’s Kappa (κ) was calculated for all domains and interviews to assess whether the two researchers coded the same response into the same domain [26,27]. Responses that were coded in different domains by the researchers were discussed to establish consensus. In instances where single domain allocation agreement could not be reached, researchers agreed that the response could be placed in both domains.

One researcher (AMP) generated statements that represented the specific beliefs from each participant’s responses that captured the core thought and continued this process for every response. A specific belief is a statement that provides detail about the perceived role of the domain in influencing the behaviour [18]. The belief statement was worded to convey a meaning that was common to multiple utterances by interview participants. When a statement was considered similar to a previously identified statement, both were coded as two instances of the same belief. Specific beliefs that centred on the same theme or were polar opposites of a theme were grouped together. This strategy was reviewed by the second researcher (RI) to ensure accurate representation of content.

Relevant domains were identified through consensus discussion between the two researchers (AMP, RI) and confirmed by a health psychologist (JJF). Briefly, three factors were considered when identifying key domains: frequency of the beliefs across interviews; presence of conflicting beliefs; and perceived strength of the beliefs impacting the behaviour. All of these factors were considered concurrently in establishing domain relevance. For example, if the belief that my emotions do not influence whether or not I order routine tests was consistently reported, it was concluded that the Emotion domain was not relevant to the behaviour. In contrast, if the majority of respondents in a study reported the belief that it is very easy to order tests then the Beliefs about capabilities domain would have been selected as relevant because of its content and the impact that it might have on physicians’ practice. Similarly Beliefs about consequences would be identified as a key domain if conflicting statements about potential consequences associated with the behaviour ranged from negative (delay or cancel patient surgery) to indifference (little or no change in patient outcome) to positive (avoid unnecessary investigation).

### Ethics

Ethics approval was obtained from the Ottawa Hospital Research Ethics Board.

## Results

### Participants

Sixteen participants, eleven anesthesiologists (9 male; 2 female) and five surgeons (all males), from community (n = 3) and academic hospitals (n = 5) in six health regions throughout Ontario were recruited to participate in the semi-structured interviews. The clinicians’ experience as a specialist ranged in years from 2.5 to 22 (mean ± SD, 10.72 ± 5.16).

### Interrater reliability

A total of 459 utterances from the 16 interviews were coded into the 12 domains. Interrater reliability for the coder across all interviews and domains had ‘almost perfect agreement’ [28] (κ = 0.84; 95% CI 0.807 to 0.878). Further, although initial interrater reliability was calculated, all disagreements between researchers were resolved through consensus.

### Key themes identified within relevant domains

Key themes emerging from the interviews with anesthesiologists and surgeons were categorised within seven theoretical domains: Social/professional role and identity, Beliefs about capabilities, Beliefs about consequences, Environmental context and resources, Social influences, Behavioural regulation, and Nature of the behaviour (Table 1).

**Table 1:** Summary of belief statements and sample quotes from anesthesiologist and surgeons assigned to the theoretical domains identified as relevant

**Domains**	**Specific belief**	**Sample quote**	**Frequency out of 16**
Social/professional role & identity	My Colleagues agree/do not agree with my opinion about Pre-op testing.	‘…I mean all my colleagues would agree with my general principles.’ (A1)	9
		‘I know my anesthesiologists….no I have had surgeries cancelled where we the patient comes in.’(S3)	
		‘Many of my colleagues have a preference for doing more pre-operative investigation than I do.’ (A6)	6
	I don't need to see an ECG or CXR to do my job.	‘Doing a chest x-ray and EKG are not part of my job per se.’ (A4)	8
		‘No, I don’t (feel it’s an obligation to order certain tests)…’ (S4)	
	I don't play a role in the ordering of tests.	‘Well I don’t make (the decision to order tests or not).’ (A11)	2
		‘So, that role being part of the team means that some of the tests will be ordered regardless of whether or not I order them.’ (A6)	
Beliefs about capabilities	It's very easy for me to order tests.	‘I pick an order sheet from the desk, I write it down and it happens…’ (A1)	16
		‘It is dead easy to order tests during a pre-op evaluation. We just write it in and that’s part of that’s part of why things are the way they are. ‘(S1)	
	I am confident that I can perform a pre-op assessment on a low-risk patient without pre-op tests.	(Are you confident that you are able to perform a pre-op evaluation for a low-risk surgery without pre-op tests?) ‘In the low-risk patient, absolutely.’ (A8)	11
		‘Definitely. (I am confident that I am able to perform a patient evaluation for a low-risk surgery without ordering pre-op tests).’ (S2)	
	It's difficult to cancel/not order because most often the tests are completed before I see the patient.	‘It is more difficult (to cancel) because some of them are ordered pre-operatively by the surgeons so the test is complete by the time you get to see the patient.’ (A4)	7
		‘Well I mean for me it’s almost impossible to cancel…because they’re done before I see them.’ (A3)	
	It's very easy for me to cancel/not order tests.	(How easy or difficult is it for you personally to cancel or order no tests as all?) ‘**Very easy.**’(A10)	7
		‘Easy (to cancel or order no tests at all).’ (S3)	
	I prefer to have routine tests for patient having general anesthesia.	‘If the patient is going to have a general anaesthetic for a lap-chole even though the surgery is low-risk, I may still feel better if I had some further investigations especially the ECG.’ (A2)	2
	I can't cancel tests that were ordered by another physician.	‘Well if another physician has ordered a test…so I can’t cancel someone else’s order.’(A4)	2
	It's difficult to cancel because it's time consuming to track down the doctor.	‘Because usually what you do if you are going to cancel a test that somebody else has ordered I think it’s your responsibility to phone the surgeon or whoever ordered the test to let him know what you are doing (right) and that takes a lot of time. You may not be able to contact people so that makes it you know often more difficult to cancel tests.’ (A6)	1
Beliefs about consequences	If tests are ordered I never/sometimes/always review them.	‘It would be expected only if it had been ordered but it certainly wouldn’t be an expectation of mine for you know for every patient.’ (A2)	5
		‘…if it’s been done then it behoves you to know the results of it. But it isn’t a requirement for me to proceed. Like I wouldn’t order it and I wouldn’t require it.’ (A3)	4
		‘In relation to low-risk surgery, I would say no (it's not expected).’ (A6)	3
		‘I know I’ll probably want to see an ECG.’ (A1)	1
	Reducing routine tests would save money.	‘Well, I mean on the positive side it’s going to save us money.’ (A4)	11
		‘The negative effects of pre-op testing, well the cost is one.’ (S3)	
	Reducing routine tests would result in little or not change in outcomes.	‘In the vast majority of patients nothing, they would just come through surgery and nobody would care.’(A2)	10
		‘If I didn’t order any at all…I don’t think it would make a heck of a lot of difference.’(S5)	
	Reducing tests may delay or cancel a patient's surgery.	‘So if somebody has a personal belief that they think every person should over 40 should have an ECG and if they arrive on the day of surgery and they haven’t gotten one and they’re going to delay surgery in order to get one, then that’s a bit of a problem.’ (A3)	9
		‘The worst thing that can happen the day of there’s a bit of surprise in the patient’s medical condition and they get cancelled, (right) that’s the worst thing that can happen.’ (S2)	
	Reducing routine testing would avoid unnecessary investigation.	‘Another positive is that it would avoid unnecessary investigations or delay in proceeding to the surgical procedure without changing the management.’ (A6)	5
		‘One of the reasons I don’t like ordering lots of tests is I get false positives and then I have to investigate them and I’m not crazy about investigating false positives especially in areas that I don’t practice in.’(S3)	
	Reducing routine tests would save patients' time.	‘I suspect that patients would like the fact that their waiting times would decrease in the pre-op consultation clinic because they don’t have to do any blood work or chest x-rays.’ (A1)	5
		‘Yes…because the negative aspects waste a patient’s time…’ (S2)	
	Reducing routine tests may result in missing an underlying condition that may complicate surgery/recovery.	‘I must say I look at everybody’s just as a matter of routine because I’ve been caught before in somebody who had electrocardiogram changes and I didn’t see it until after I put the patient to sleep and that was when I was a junior resident. And so from then on I’ve been very wary about looking at the electrocardiogram.’ (A7)	4
		‘I mean the issue at that point is you know is it safe to do the surgery, is there some unexpected finding that means we shouldn’t be doing the surgery on that basis or is there something that would change our decision.’ (S1)	
	Tests are ordered routinely because there pretty cheap.	‘…it just doesn’t cost me anything, I’ll do it.’ (A6)	2
		‘I mean…personally I don’t see much of an issue in doing a non-invasive test like an EKG which would also be relatively low expense as well.’ (S4)	
Environmental context and resources	Time is/is not a factor in my decision to not order tests.	‘I wouldn’t say that (time constraints) ever influenced me in what test to order, if I ever thought something was necessary I would order it.’ (A1) ‘Not really…(there aren't any competing tasks or time constraints).’ (S2)	7
		‘Time efficiency…(is important). And you know as long as clinics are that busy, you have to focus on flow through, so I sort of view ECGs as pretty cheap tests all things considered.’ (A9)	5
		‘So there’s no question that time … [play a big role] mainly just kind of default to what you’ve always done.’ (S1)	
	The Medical directive at this hospital dictates that no routine testing/routine testing for low-risk surgeries.	‘The only tests that happen are through medical directives.’ (A6)	7
		‘I mean I complete those forms.. just tick the box, it couldn’t be easier, and then put in some blood work and chest x-ray and cardiogram if those are, you know, flip through my mind in the 2 or 3 seconds. (S1)	
		‘…we have mandated that in this hospital no pre-operative testing is done.’ (A10)	3
	There is nothing in my clinic environment that influences whether I order tests or not.	‘There’s no impediment to us ordering these tests and having them done pre-operatively.’ (A8)	9
		‘Not really - (there anything that impedes or advances)’ (S4)	
	The medical directive at our hospital is that the surgeon orders the tests.	‘Not typically true, I mean our department has developed a guideline that’s it’s followed. The guideline is the surgeons if they order a test, if any test is ordered will be done. If there’s no test ordered, the patient has the guidelines followed.’ (A8)	3
		‘Yes so we would in our institution typically the surgeons would have ticked off the order sheet.’ (A11)	
Social influences	The opinions of others do/do not influence my decision to order routine tests.	Might the views/opinions of others affect you ordering certain tests for a pre-op evaluation for patient having a low-risk surgery? ‘It doesn’t affect me.’ (A5)	11
		‘I find that yes I would listen to them and say okay let’s order it and see what it shows and maybe I’ll learn something from it as well.’ (S2)	
		‘…when I’ve signed that nobody’s going to say oh he doesn’t know what he’s talking about. They’re going to say oh geez, he doesn’t know what he’s talking about but we’d better do it anyway.’ (S1)	4
		‘Uh only the anesthetist (would influence whether or not I order certain tests).’ (S4)	
	Patient emotions do/do not influence whether or not I order routine tests.	‘The nurse will sometimes say in the pre-op clinic thing that a patient is highly anxious but that would never make me do further investigations.’ (A3)	12
		‘No - patient emotions don't influence whether or not I order certain tests.’ (S5)	
		‘They do. You know I’ve got a philosophy to tell patients they know their body better than I do…’ (S3)	3
	I order test I feel are unnecessary because my conservative colleague may be in the OR the day of the surgery and want to see the routine test that I would not.	‘It means that I may not be the anaesthetist doing the case. So I have to not only make a judgement as to what would be appropriate for me, but also what might be appropriate for my colleague as well doing the anaesthetic.’ (A1)	6
		‘…because we see patients for each other so.you always have to think about what each of your colleagues may want and everybody has a little bit different practice… based on my colleagues I might be inclined to order a few more tests than I would if I knew that I was going to do the anaesthetic…’ (A9)	
		.’.I might anticipate that the anaesthetist would want particular tests, or a report that anaesthetists in general might want a particular test.’ (S4)	3
	I'm reluctant to cancel test ordered by other physicians.	‘But it is one of the issues because of course, if a surgeon ordered it, I’m somewhat more reluctant to cancel one of their tests even though I don’t feel it’s that necessary.’ (A4)	4
		‘Sometimes they are ordered and then (I) might be reluctant to cancel some of the tests because I’m not privy to the thought process initially went through the other individual’s mind and so…I may hesitate because I think well does he have a good reason for ordering this test that I’m not aware of.’ (A2)	
	Because you work with a group we have to come to an agreement as to what test are required.	‘…the important thing is you need to decide as a group when you work as a group you have to decide what everybody agrees upon for what tests are required.’(A7)	3
		‘So I think that they’ve been quite good in supporting you know their colleagues that way. So most of the time that works well.’ (S3)	
Behavioural regulation	We need policy that takes the test ordering out of the hands of the surgeons.	‘Right now we don’t have a medical director of our pre-op clinic and that’s probably something, you need someone dedicated to the role to address these kinds of questions.’ (A7)	7
		‘I think they would be evaluated during their pre-operative assessment or that assessment would either be done by an anaesthesiologist’ (A10)	
		‘Well probably take it largely out of the hands of individual surgeons and make it a matter of policy.’ (S1)	
	There needs to be better evidence that show testing isn't necessary in low-risk patients.	‘The better way probably which is accumulated evidence suggests that the tests aren’t really necessary in the low-risk the low the low-risk patient undergoing low-risk surgery.’ (A5)	5
		‘I think if we had more data to support the fact that testing is not necessary that would go a long way.’ (A3)	
Nature of the behaviour	I typically do/do not review tests when ordered.	‘In relation to low-risk surgery, I would say no (reviewing an CXR or ECG is not an expected part of my check).’ (A1)	7
		‘No. (I don’t typically review a CXR or ECG before my patient’s operation?) (S4)	
		‘If ordered, yes.(I review tests)’ (A6)	6
		‘It would be expected only if it had been ordered.’ (A2)	
	I typically do/do not order tests.	‘Yeah, for these patients I would not, for the true low-risk patients I would not order the tests automatically.’ (A3)	7
		‘I’m actually one of the people who is in favour of not ordering tests that are not needed…in a low-risk patient.’ (S4)	
		‘The default is…the default is to order…’ (A4)	3
	Typically all tests are order before I see the patient.	‘On a standard basis they would be ordered by the surgeon’s office.’ (A5)	9

Note: *‘*A#*’* indicates sample quote by anesthesiologist *‘*S#*’* indicates sample quote by surgeon.

While both groups felt that they did not need to order or review a CXR or ECG to adequately do their job when performing a low-risk surgical procedure on a healthy patient, they made conflicting comments as to who exactly was responsible for ordering the pre-operative tests and responses within each professional group varied (Social/professional role and identity). For example, several anesthesiologists stated that they should have complete autonomy as to what tests should be ordered whereas others noted that within their hospital it was not their responsibility to order the pre-operative tests (Nature of the behaviour, Social/professional role and identity, Environmental context and resources). Conversely, some surgeons noted that pre-operative test ordering was the responsibility of the anesthesiologists, while others mentioned that they were the most responsible physician in the operating room and as such had the ultimate responsibility to understand the whole picture (Social/professional role and identity).

Both anesthesiologist and surgeons reported that it was very easy to order any pre-operative test they wanted—they just ticked a box on the admitting forms (Beliefs about capabilities, Environmental context and resources). However, anesthesiologists noted that there was a problem with their inability to cancel tests ordered by the attending surgeon, because they did not know the initial reasoning behind the surgeon ordering the test (Beliefs about capabilities, Social influences). Further, they mentioned that often when surgeons ordered pre-operative tests, the tests were usually completed before the anesthesiologist sees the patient (Beliefs about capabilities, Environmental context and resources).

Interestingly, anesthesiologists noted that they often ordered tests they did not think necessary to prevent a cancelled surgery if those tests were required by a colleague with different preferences regarding testing for anesthesia management (Beliefs about capabilities, Social influences, Beliefs about consequences). They also noted that because they work with a team there is often an understanding among their colleagues as to what tests are required and they tend to be conservative and order more, to cater for majority views (Social influences, Beliefs about capabilities). The surgeons gave conflicting information about colleague influence. They stated that they rely on the anesthesiologists to order the necessary pre-operative tests and listen to their other team member before making a decision regarding what tests to order, but mentioned that no one would question their request for certain tests; staff would just follow the surgeons’ requests (Social influences).

Both surgeons and anesthesiologists reported variable practice in their personal review of pre-operative tests before commencing with anesthesia and surgery (Nature of the behaviour). There were also conflicting comments about the potential consequences associated with reducing testing (Beliefs about consequences). Both anesthesiologist and surgeons agreed that routine tests are a waste of time and money, unnecessary, and rarely provide any useful information. They stated that routine testing may result in false positives that require investigation, and reducing test ordering would avoid unnecessary investigations and delays. Yet, they also mentioned that routine testing saves patients' time and if routine tests are not ordered, a patient's surgery may get cancelled or miss an underlying condition that may complicate surgery and ensures the patient is fit for the surgery.

Both anesthesiologists and surgeons identified factors within their environment that affected their decision to order pre-operative tests (Environmental context and resources). There was considerable disagreement as to whether time constraint was a factor in test ordering practice.

There were also reports of a gap between their motivation and practice (Behavioural regulation). Both anesthesiologists and surgeons mentioned if hospitals made sure that all pre-operative testing was conducted by only anesthesiologists and took the ordering out of the hands of the surgeons, unnecessary routine testing could be reduced.

### Domains reported not relevant

Five domains appeared to be less relevant: knowledge, motivation and goals, skills, memory, attention and decision processes, emotion (Table 2). The majority of anesthesiologists and surgeons were aware of the guidelines and knew they were supported by evidence-based research (Knowledge). Both groups reported that they didn’t feel obligated to order tests for anesthesia management for a low-risk surgery, and some stated that routinely ordering tests was not an important part of their pre-operative evaluation (Motivation and goals). In addition, they stated that there was no set of specific skills required to order pre-operative test and that nurses, general practitioners, and other physicians (internists) can order them if appropriately trained (Skills). When asked about their Memory, attention, and decision processes, anesthesiologist and surgeons stated that they focus mainly on patient history and medical condition when deciding what tests may be required at the time of a patient’s surgery. Further, all respondents interviewed stated that their own emotions would not influence whether they ordered pre-operative tests or not (Emotion).

**Table 2:** Summary of belief statements and sample quotes from anesthesiologist and surgeons assigned to the theoretical domains identified as not relevant

**Domains**	**Specific belief**	**Sample quote**	**Frequency out of 16**
Knowledge	I am aware of guidelines. (provincial/national)	‘Yes, so there are guidelines from the Canadian Anaesthesia Society and then various bodies around the world have published guideline…for pre-operative testing.’ (A5) ‘I can’t recite you any specific guidelines but I’ve heard that there are some standards that way either from talking to anaesthetists…so yes there are some guidelines but I can’t tell you specifically.’ (S5)	15
Skills	As long as you're adequately trained to take a pre-op assessment you are skilled enough to order ‘routine’ tests.	‘So I think experience in pre-operative assessment clinics during training and some exposure to surgery or understanding of it… ‘(A2) ‘At a minimal you should have training as a nurse…in terms of some specialized training to screen patients.’ (A8) .’.in general, you know particularly with you know a low-risk population and a low-risk operation, I thought a person with experience, training and interest so on, could probably do very well.’ (S1)	16
Motivation and goals	I, personally, do not feel I need to order routine tests.	‘No….it’s something that I don’t think needs to be done.’ (A7) ‘No…(it's not something I need to do).’(S1)	14
	Routinely ordering tests is not an important part of my pre-op evaluation.	‘When it’s necessary it’s very important but overall I think most of the time it’s unnecessary.’ (A6) ‘It’s not important (to perform Pre-op tests in your pre-op evaluation of a pt. having a low-risk surgical procedure).’ (S3)	9
Memory, attention & decision processes	My decision to order or not order tests is based on patient history and medical condition.	‘I would only order them if I felt that there was some sort of medical issue that needed to be addressed. Yeah that’s it.’ (A3) ‘My pre-operative evaluations are primarily related to their surgical condition…’ (S5)	16
Emotion	My emotions do not influence whether or not I order routine tests.	Does not ordering tests in a pre-op evaluation for patient having a low-risk surgery evoke worry or concern in you? **‘No it wouldn’t.’** (A1) ‘If they do not need it and I am not ordering it, I’m not at all concerned about it, no.’ (S4)	16

Note: *‘*A#*’* indicates sample quote by anesthesiologist *‘*S#*’* indicates sample quote by surgeon.

## Discussion

This study applied the TDF [20] to help understand the influences of pre-operative test ordering practices for anesthesia management in healthy patients by anesthesiologists and surgeons. The results show that the most frequently mentioned influences on the clinicians’ test ordering practice were categorised primarily in the Social/professional role and identity, Beliefs about capabilities, Beliefs about consequences, Environmental context and resources, and Social influences domains, and centred around two key issues. First, the lack of clarity by hospital management and lack of written policies as to who was ultimately responsible for ordering the tests (Social/professional role and identity, and Environmental context and resources) is a considerable factor influencing whether or not they order routine pre-operative tests. Respondents reported that hospitals commonly either failed to identify which group was specifically responsible for test ordering or identified surgeons as the group responsible for test ordering. Further, the existence of hospital directives varied from hospital to hospital throughout the province (Environmental context and resources). The finding that surgeons often order pre-operative tests according to hospital policies seems counterintuitive because the Canadian Anesthesiologists’ Society is the professional body making the recommendations and state that policies regarding pre-anesthetic assessment should be established by the department of anesthesia [7]. Yet, the anesthesiologists and surgeons interviewed report this finding as accurate and is further supported by evidence documented by Bryson *et al.*[29]. The likelihood that an alternative professional group would review another’s guidelines is rare because they struggle to keep up-to-date with their own ever-changing evidence-based practice. So how do we ensure that those responsible obtain the best and most current evidence? A directive by hospital management that is supported by the professional groups involved, as to which group holds the role and responsible for ordering the tests required for anesthesia management would likely reduce confusion and encourage greater consistency in test ordering practices.

Second, evidence of the inter-professional influences among the attending surgeon performing the surgery, the anesthesiologist at pre-admission ordering the tests, and attending anesthesiologist providing intraoperative care was reported by the vast majority of respondents (Social/professional role and identity, Beliefs about capabilities, Belief about consequences, and Social influences). The lack of clarity about who is responsible for routine test ordering appears to lead to a propensity to order tests *‘*just in case*’* they are expected by another colleague. A surgeon may order the tests *‘*in case*’* the attending anesthesiologist needs it and in hopes that the patient will move smoothly through the pre-admission assessment process. The anesthesiologist who sees the patient prior to the surgery orders the tests *‘*in case*’* the attending anesthesiologist needs them and could not cancel tests ordered by the surgeon because they have not identified the reason for ordering the tests. Furthermore, the anesthesiologists interviewed reported they seldom reviewed test results when caring for low-risk patients in the operating room. The interesting thing about the team influence is that although anesthesiologists and surgeons greatly influence whether pre-operative test are ordered by another team member, these clinicians rarely have direct contact with one another and communication is difficult. A study by Lingard *et al.* examined intraoperative communication in a surgical team comprising surgeons, nurses, anesthesiologists, and trainees [30]. They found marked differences in the professionals’ perceptions around issues of role authority, motivation, and value with respect to communication among team members. Although their study looked at four professional groups, their findings are consistent with ours in identifying a problem in the lack of clarity relating to roles of surgeons and anesthesiologists. They suggest that communications of these team members are probably motivated by some combination of concern for the patient, the day’s schedule, ethical issues, economic implications, and many other factors [30], an idea that is reflected in our finding of professionals ordering test just *‘*in case*’* the tests are needed. Further, communication with respect to pre-operative testing is additionally complicated by the surgeons’ and anesthesiologists’ separation by time and space.

This study is one of the first to attempt to examine why anesthesiologists and surgeons order routine pre-operative tests when no clinical indicators exist. There has been a large body of work reporting pre-operative testing practices [2,4,6,10,31-33]. However, few attempt to explain why clinicians do one thing when the guidelines recommend another with respect to test ordering for anesthesia management [7]. A systematic review by Munro *et al.* reported that the value of pre-operative ECGs in predicting postoperative cardiac complications seems to be very small, and the indirect evidence suggests that routinely recorded pre-operative ECGs as a baseline measure are likely to be of little or no value [34]. Further the anesthesiologists and surgeons interviewed appear to lend credence to this report. Yet, reports continue to document unnecessary routine test ordering [2,4,6,10,31-33], and we have attempted to ask those clinicians involved why unnecessary tests for anesthesia management continue to be ordered. Bryson *et al.* was the only paper reviewed to suggest a need to change *‘*established behaviour*’* that should include not only anesthesiologists but surgical colleagues and clinic personnel [10]. By examining the views of the clinical decision makers (anesthesiologists and surgeons) in a theory-based systematic manner, we have identified the theoretical domains we propose best predict pre-operative test ordering for anesthesia management when assessing healthy patients undergoing low-risk surgeries.

Seven domains were considered potentially important for changing test-ordering behaviour (Social/professional role and identity, Beliefs about capabilities, Beliefs about consequences, Environmental context and resources, Social influences, Behavioural regulation, Nature of the behaviour), while five were consistently identified as not relevant (Knowledge, Skills, Emotion, Motivation and goals, and Memory, attention and decision processes). Of the seven identified the five that appeared to be the most influential, based on the frequency of utterances coded and content of the responses, were Social/professional role and identity, Beliefs about capabilities, Beliefs about consequences, Environmental context and resources, and Social influences. The TDF is a relatively new framework that attempts to help understand clinical behaviour from a psychological perspective. Previous attempts to understand clinicans’ behaviour has either been atheoretical [11-14] or have used a limited number of theories [35-37] with varying effectiveness. Ideally, researchers should have ready access to a definitive set of theoretical explanations of behaviour change and a means of identifying which are relevant to particular contexts [20]. The TDF allow for a categorisation of respondents’ views in a theoretically-based systematic way that attempts to encompass a broad range of psychology theories without favouring a specific one.

While this study has provided valuable insight into the factors that may influence routine test ordering practices, there were several limitations. It is possible that saturation could have been prematurely reached if participants recommended interviewing others with similar opinions. In an attempt to avoid this, one of the criteria used in our purposive sampling was to ask the participants to recommend additional anesthesiologists with differing opinions. Subsequently, our results show that there was evidence of differing opinions from the anesthesiologists and surgeons about order test routinely ordered for anesthesia management.

Identification of themes does not provide evidence of the actual influences on clinical practice. These are merely clinicians’ views about what might influence their test ordering behaviour. Although interview studies are required in the exploratory stages of research in this field, different research designs would be required to establish which of these factors could be key to changing practice.

In this study the interview guide used a combination of questions that elicited descriptive and diagnostic responses (*e.g.*, *‘*What thought processes might guide your decision to order pre-operative test for a patient having a low-risk surgery?*’* is descriptive, whereas *‘*Are you confident that you are able to perform a pre-operative evaluation for a low-risk surgery without pre-operative tests?*’* is diagnostic). It thus required further interpretation by the research team to decide whether a descriptive response represented a barrier to changing practice. For studies that use the TDF for problem analysis, it may be preferable to use more questions of the diagnostic kind.

Our study has shown that in various hospitals across the province of Ontario anesthesiologists are often not the professional responsible for ordering the pre-operative tests, even though the Canadian Anesthesiologists’ Society has published guidelines directing this aspect of perioperative care. Interviewing surgeons in addition to anesthesiologists strengthened our findings because it gave us the perspectives from both key professional groups responsible for ordering pre-operative test. It also identified the link between attending surgeon, assessing anesthesiologist, and attending anesthesiologist as an important social influence of pre-operative test ordering. Additional strength in our findings was that even though the two groups differ in their role in the care of patients, their responses around pre-operative test order practice largely converged. Both groups throughout the province repeatedly identified the same issues of concern. Recently, there have been a numbers of studies examining the inter-professional dynamics within a team of healthcare providers [30,38-41] but further work is necessary to better understand the inter-professional dynamics of a healthcare team. Developing an intervention that would take into consideration the roles of all personnel involved in the care of a patient undergoing low-risk surgery has the greatest likelihood of being successful and should be developed using the domains identified in this study; in particular social/professional role and identity, beliefs about consequences, environmental context and resources and social influence.

## Conclusion

This study is one of the first to attempt to examine why anesthesiologists and surgeons order routine pre-operative tests. Our results identified potential influences, as defined by the TDF, upon test ordering behaviour of anaesthesiologists and surgeons when clinical indictors are not present. It offers a possible explanation to the test ordering differences reported by Hux *et al.*[8] and may help explain why routine tests are continually ordered when evidence shows their lack of value for perioperative management [2,4,29,32]. Our findings can be used to develop a confirmatory predictive study to further explore determinants of routine pre-operative test order practice by developing a questionnaire for the key professionals based on the domains and content of the interviews. In addition, the results can be used to develop an intervention using intervention mapping directly from the domains [42]. By using the TDF, our study provides a theory-driven basis to identify predictors of clinician behaviour as well as generate possible interventions for the reduction of unnecessary pre-operative tests routinely ordered for anesthesia management.

## Abbreviations

TDF: Theoretical Domains Framework; RCT: Randomized Controlled Trial; ECGs: Electrocardiographs; CXRs: Chest X-rays; A#: Anesthesiologist; S#: Surgeon.

## Competing interest

Martin Eccles is Co-Editor in Chief of *Implementation Science*; Anne Sales is an associate editor of *Implementation Science*; Jeremy Grimshaw and France Légaré are members of the Editorial Board of *Implementation Science*.

## Authors' contributions

JMG, JJF and the Canada PRIME Plus team conceived the study. AMP, JMG contributed to the daily running of the study. JJF oversaw the analysis which was conducted by AMP and RI. GLB provided content expertise in the filed of Anesthesiology. AMP wrote the manuscript and the authors listed commented on the sequential drafts of the paper and agreed upon the final manuscript. All authors read and approved the final manuscript.

## Authors' information

JMG holds a Canada Research Chair in Health Knowledge Transfer and Uptake. The Canada PRIME Plus team is an international collaboration of researchers consisting of health services researchers, health psychologists and statisticians.

## Supplementary Material

Additional file 1 Interview topic guide for Anesthesiologists and Surgeons. Click here for file
