# Supplementary material for: Anesthesiologists’ and surgeons’ perceptions about routine pre-operative testing in low-risk patients: application of the Theoretical Domains Framework (TDF) to identify factors that influence physicians’ decisions to order pre-operative tests
Source: Implement Sci. 2012 Jun 9;7:52. doi: 10.1186/1748-5908-7-52 (PMC3522997; doi:10.1186/1748-5908-7-52)
Supplement: Additional file 1 — Interview topic guide for Anesthesiologists and Surgeons. [file 1748-5908-7-52-S1.pdf]

Additional File #1: Interview topic guide for Anesthesiologists and Surgeons.

Semi-Structured Interview Schedule for Pre-Op Evaluations by Anaesthesiologists / Surgeons in Low-risk Surgeries

Introduction: *The general aim of the interview is to help us understand how you manage your pre-op evaluation. We want to know what influences you when you are face with the decision to order certain tests or not. There is no right or wrong answers here; we are trying to understand how different clinicians approach this issue, so please answer frankly.*

I'd like to start with some basic question about your practice:

1. What type of setting do you practice in? (community vs. academic)
2. How many years have you practiced?
3. Do you practice or work closely with residents?
4. Would you describe your pre-op evaluation "process" for a patient undergoing a low-risk surgery? (*Environmental Context)*
5. As an anaesthesiologist, how often do you come across patients undergoing low-risk surgeries? Percentage *(nature of the behaviour*)

Thank you.

For the rest of the interview, I have some slightly more specific questions about what influences your decision regarding pre-operative testing for patients undergoing low-risk surgeries. Some questions may seem repetitive, but please bear with me as the questions are derived from different theories of human behaviour and we are trying to figure out which theories best apply in this area.

Are you ready to get started?

Set the scene re: I'm asking about "your pre-op evaluations for patients undergoing low-risk surgeries" – I'd like you to think about that for a moment…

When I say low-risk surgeries, I'm thinking about those patients having Knee Laproscopy, Laparoscopic Cholescystectomy, Cataract Removal and Lens Replacement; surgeries like those. In addition when I mention pre-op tests, I'd like you to focus on CXRs and ECGs.

Knowledge

6. Are you aware of any guidelines (national, provincial or institutional) about pre-operative testing?
7. What are your thoughts about pre-op testing guidelines in general? (Prompt: Professional autonomy?)
8. Do you believe it to be evidence based? What is your interpretation of the evidence?

Skills

9. How much expertise or experience do you think one needs to have to effectively perform a pre-op evaluation?

Nature of the Behaviour

10. Is a review of a CXR or ECG an expected part of your Pre-op check?
11. Would you be comfortable proceeding without testing? WHY or Why not?

Memory, Attention and Decision Processes

12. What thought processes might guide your decision to order pre-op test for a patient having a low-risk surgery? (Prompt: "What goes through your mind?")
13. Is **not ordering** pre-op tests an automatic part of your job or is it something you take time to think about with patients having low-risk surgeries? (Prompt routine, automatic)
14. Is it typically an easy or difficult decision to make? (Prompt: Weigh pros and cons etc.)

Social/Professional role & identity

15. If you are doing a pre-op evaluation and you don't see an ECG or CXR, do you think you're doing your job? (prompt are these two test part of an effective evaluation?)
16. Is there anything in your professional role that influences your decision to order or not order certain tests for a patient having a low-risk surgery? (Prompt: professional training, a protocol, an order set, other technologies)

Environmental Context & Resources

17. What aspects of your clinical environment (physical vs. resource factor) influence whether or not you are able to ordering tests for a pre-op evaluation for patient having a low-risk surgery?
18. Are there any competing tasks or time constraints that might influence whether or not you order tests for a pre-op evaluation for patient having a low-risk surgery?
19. What would help you overcome these problems/difficulties? (Prompt: skills training in medical curriculum, communication techniques, formal training programs, CME, educational material on line or by mail)

Beliefs about Capabilities

20. How easy or difficult is it for you personally to order tests during a pre-op evaluation?
21. How easy or difficult is it for you personally to cancel or order no tests as all?
22. Are you confident that you are able to perform a pre-op evaluation for a low-risk surgery without pre-op tests?

Social Influences

23. Would any other team members influence whether or not you order certain tests for a pre-op evaluation for patient having a low-risk surgery? (Prompt: who else; Other clinicians; medical staff including nurses and residents/fellows; relatives; surgeons test orders)
24. How might the views/opinions of others affect you ordering certain tests for a pre-op evaluation for patient having a low-risk surgery?
25. Do your colleagues generally agree with you on this issue?

Emotion

26. Do patient emotions ever influence whether or not you order certain tests for a pre-op evaluation for patient having a low-risk surgery? Explain
27. Does not ordering tests in a pre-op evaluation for patient having a low-risk surgery evoke worry or concern in you?

Beliefs About Consequences

28. Do you believe the costs of pre-operative testing are worth the benefit in low-risk surgeries? Why or why not?
29. What do you think will happen if you don't order a ECGs or CXR during you pre-op evaluation, both positive and negative? (*prompt: to patients, to colleagues, yourself, short and long term)*

Motivation and Goals

30. In your pre-op evaluation of a patient having a low risk surgery, do you plan to routinely order ECG and CXRs? - Is it something you feel you need to do?
31. How important is it to you to perform Pre-op tests in your pre-op evaluation of a pt. having a low risk surgical procedure?
32. What would be an incentive for you to reduce the number of per-op tests you order when evaluating patients for low-risk surgeries? (Prompt: goals within yourself? external?)

Behavioural Regulation

33. When faced with a patient preparing for a low-risk surgery, would ***not ordering*** pre-op tests be something you would usually do?
34. What would you, personally, have to do to decrease the number of pre-op tests you order for a patient having low-risk surgery?

> *Follow-up* In an ideal world, where anything is possible, what would see as best process for ordering pre-operative tests for a patient having a low-risk surgery?

35. If you wanted to implement changes in your own practice (individual/team setting/practice setting) to discourage pre-op testing for low risk surgeries, what would be some ways to do this? (Prompt: Role of Protocol or guidelines)

That's all the questions I have for you; has anything occurred to you about this topic that we haven't asked about?

Thank you!
